# Continuous Phosphate Removal and Recovery Using a Calcium Silicate Hydrate Composite Monolithic Cryogel Column

**DOI:** 10.3390/polym15030539

**Published:** 2023-01-20

**Authors:** Chanadda Phawachalotorn, Worawit Wongniramaikul, Tarawee Taweekarn, Bussakorn Kleangklao, Wachiraporn Pisitaro, Wadcharawadee Limsakul, Wilasinee Sriprom, Wanchitra Towanlong, Aree Choodum

**Affiliations:** 1 King Mongkut’s Institute of Technology Ladkrabang, Prince of Chumphon Campus, Chumphon 86160, Thailand; 2 Integrated Science and Technology Research Center, Faculty of Technology and Environment, Prince of Songkla University, Phuket Campus, Kathu, Phuket 83120, Thailand; 3 Office of Scientific Instrument and Testing, Prince of Songkla University, Songkhla 90110, Thailand

**Keywords:** starch cryogel, calcium silicate hydrate, continuous flow adsorption, phosphate removal

## Abstract

Toward the development of a practical and green approach for removing phosphate from water, a monolithic cryogel based on starch and calcium silicate hydrate (Cry–CSH) was employed as a phosphate adsorbent in a continuous flow system for the first time. The influence of flow rate, initial phosphate concentration, and adsorbent height on the adsorption efficiency was investigated. As the rate of flow and the initial concentration of phosphate increased, the total quantity of adsorbed phosphate dropped; however, the performance of the column was greatly enhanced by an increase in adsorbent height. The experimental data fit the Adams–Bohart model better than the Thomas and Yoon–Nelson models at the beginning of the adsorption process. To evaluate its applicability, the continuous flow system based on the monolithic Cry–CSH column was applied for the removal of phosphate from the discharge effluent of the Patong Municipality Wastewater Treatment Plant (Phuket, Thailand), achieving an excellent total adsorption of 94.61%.

## 1. Introduction

In the past few years, the risk of eutrophication of water bodies due to excessive phosphate emissions from natural sources and human activities has become a pressing concern [1]. Phosphate (soluble reactive phosphorus) is a critical nutrient for the growth of phytoplankton and algae [2,3,4,5]. At concentrations between 0.025 and 0.03 mg L^−1^ [6] or higher than 0.02 mg L^−1^ [7,8], phosphate induces the eutrophication of water bodies, resulting in the deterioration of water quality [9,10]. Thus, controlling the phosphate concentration in effluents prior to their discharge into water bodies is imperative for reducing pollution. Numerous methodologies for removing phosphate from effluents have been developed, including coagulation [11], biological treatment [12], adsorption [13], and chemical precipitation [14]. Adsorption is one of the most appropriate technologies due to its simplicity of use, high removal effectiveness, and economic affordability [15,16,17,18]. To adsorb phosphate from aqueous solutions, various materials have been investigated in batch experiments, such as chitosan [19], activated carbon [20,21], silica gel [22], clays [4,23], zeolites [24,25], carbon nanotubes [26,27], coal [28,29], and fly ash [30,31].

Recently, calcium silicate hydrate (CSH) has emerged as an adsorptive material exhibiting great potential for phosphate removal with respect to other adsorbents [1,2,10,32,33,34,35]. In addition, CSH can be employed for phosphate recovery in biological wastewater treatment as it serves as a calcium ion donor, seed crystal, and pH regulator simultaneously [35,36,37]. However, CSH materials are generally used as fine powders that can be lost to effluents, resulting in a reduction in adsorption efficiency [38]. To prevent the loss of phosphate-removing powders, polyvinyl alcohol (PVA) is commonly utilized as a binding carrier because of its chemical stability, high mechanical strength, and nontoxicity [39,40]. Recently, a starch-based monolithic cryogel composited with CSH (Cry–CSH) has been reported to remove phosphate with >98% efficiency in a batch method [10].

Herein, to evaluate the practicality of this novel adsorbent, the Cry–CSH monolith was used for the removal of phosphate using a continuous flow adsorption system. Various factors affecting the column efficiency, such as the flow rate, the initial phosphate concentration, and the adsorbent height, were evaluated. Moreover, the column dynamics were investigated using theoretical breakthrough curve models, i.e., the Thomas, Adams–Bohart, and Yoon–Nelson models. Natural phosphate-containing wastewater from Pak Bang Canal in the Patong district (Phuket, Thailand) was directly monitored using this continuous flow adsorption system to assess its applicability. Thus, this work demonstrates an interesting tool to achieve Target 14.1 (to prevent and significantly reduce nutrient pollution by 2025) for the Life Below Water Sustainable Development Goal (Goal 14) published by the UN [41].

## 2. Materials and Methods

### 2.1. Materials

Na_2_HPO_4_ (analytical reagent grade) was purchased from Ajax Finechem (Sydney, Australia) and used to generate a phosphate stock solution (100 mg L^−1^). C_6_H_8_O_6_ (analytical reagent grade) was purchased from Fisher Scientific (Leicestershire, UK). (NH_4_)_6_Mo_7_O_24_·4H_2_O and C_8_H_4_K_2_O_12_Sb_2_·3H_2_O were supplied by Carlo Erba (Val-de-Reuil, France). CaCl_2_ (99%) and Na_2_SiO_3_ were supplied by Loba (Mumbai, India). Cassava starch (Jaydee Brand, Nakhon Pathom, Thailand) and rice flour (Erawan Brand, Nakhon Pathom, Thailand) were acquired from a grocery store in Phuket, Thailand. According to the Thai Agricultural Standard (TAS 4000-2003), a spectrophotometer was used to determine their amylose content, which was found to be 27% and 23%, respectively (in-house method TE-PH-021 based on Quality and Testing of Thai Horn Mali Rice, 2004, Department of Agriculture, Thailand). Each standard solution was formed with ultrapure water that was filtered in a water purification system (Merck, Darmstadt, Germany).

### 2.2. Preparation of the Cry–CSH Monolithic Column

Cry–CSH was prepared according to our previous study via a freeze–thaw process [10]. Briefly, a saturated calcium hydroxide solution (130 mL) was used to disperse 12.5 g of rice flour and 3.75 g of tapioca starch. The mixture was then gradually heated from 60 °C to 200 °C over a period of 1.5 h to produce a clear gelatinized starch. CSH (4.5 g) synthesized via a rapid ultrasound-assisted sol–gel method [10,35] was then homogeneously mixed with 60 g of the gelatinized starch. Then, the mixture was filled into a 300 mL plastic syringe and frozen at −20 °C for 24 h. The obtained monolith was thawed at ambient temperature, and the freeze–thaw process was replicated three times. The as-obtained Cry–CSH was removed from the syringe and cut into 1 cm-long pieces. The resulting cryogel monoliths were soaked in 95% ethanol for one day, dried in an oven at 100 °C until achieving stable weights, and then stored in a desiccator inside airtight plastic bags until further use.

### 2.3. Characterization of the Monolithic Cry–CSH Column

Field emission scanning electron microscopy (FESEM; FEI, Brno, Czech Republic) was used to investigate the Cry–CSH’s morphology both before and after phosphate adsorption. Using monochromatic Cu Kα radiation, X-ray diffraction (XRD) patterns were produced using an X-ray diffractometer (Empyrean, PANalytical, The Netherlands). Fourier transform infrared spectroscopy (FT–IR; Bruker, Germany) was used to examine the functional groups of adsorptive materials using the ATR technique and KBr pellets at 4000–400 cm^−1^.

### 2.4. Experimental Setup for Continuous Flow Adsorption Study

**Figure 1:**
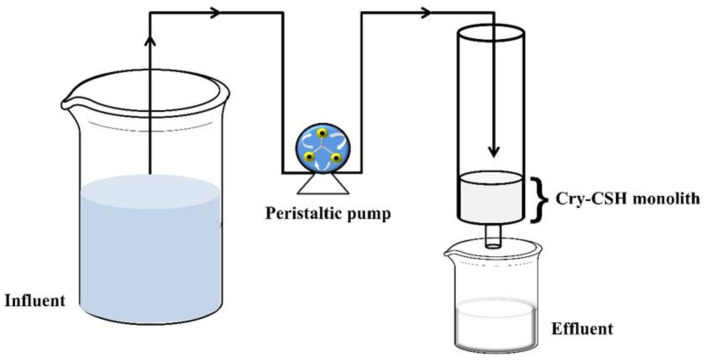
Schematic flowchart of an experiment design for column absorption study.

Figure 1 shows a continuous flow adsorption study performed in a plastic tube with an inner diameter of 5.0 cm filled with a monolithic Cry–CSH adsorbent column. A peristaltic pump was used to feed phosphate solutions through the monolithic column in down-flow mode. All column tests were conducted at ambient temperature to represent environmentally relevant conditions. Periodically, samples of the effluent were taken and examined to determine the concentration of phosphate ions still present in the water using an ultraviolet spectrophotometer according to the standard ascorbic acid method [42]. The column adsorption was performed until phosphate concentration in the influent and effluent remained unchanged.

The following three parameters affecting the adsorption efficiency and capacity were investigated under the present experimental design: flow rate (5.0, 7.5, 10, and 15 mL min^−1^), initial phosphate concentration (1.0, 2.0, and 4.0 mg L^−1^), and adsorbent height (1.0 and 2.0 cm).

### 2.5. Column Data Analysis

Generally, the performance of a column in phosphate removal from aqueous media can be assessed using breakthrough curves, whose shape determines the column operation and dynamic response [43]. Breakthrough curves can be obtained from a series of column adsorption experimental data by plotting the time-dependent ratio of the effluent phosphate concentration at time *t* to the influent phosphate concentration (*C*_t_/*C*_0_) for a given adsorbent height. Breakthrough time (*t*_b_) is defined as the period when the phosphate concentration in the effluent (*C*_t_) approaches 10% of the influent concentration (*C*_0_), i.e., *C*_t_/*C*_0_ = 0.10. In addition, the exhaustion time (*t*_e_) is the moment when *C*_t_ reaches 90% of *C*_0_ (*C*_t_/*C*_0_ = 0.90) [44,45]. The effluent volume (*V*_eff_; mL) can be determined using Equation (1) as follows:

(1) $$V_{\mathrm{eff}}=Q\cdot t_{\mathrm{total}}$$

where *Q* is the volumetric flow rate (mL min^−1^) and *t*_total_ (min) is the operation time at which the adsorption equilibrium of the column reaches saturation.

The total adsorption capacity or the total amount of adsorbed-phosphate quantity (*q*_total_; mg) in the column for a particular initial concentration and flow rate (*Q*) can be estimated from Equation (2) [46]:

(2) $$q_{\mathrm{total}}=\frac{Q}{1000}\int_{t=0}^{{t=t}_{\mathrm{total}}}C_{\mathrm{ad}\;}dt$$

where *C*_ad_ (mg L^−1^) is the adsorbed-phosphate concentration and can be obtained using the relation *C*_ad_ = *C*_0_ − *C*_t_ [43]. The equilibrium phosphate uptake or saturation loading capacity (*q*_e_; mg g^−1^) of the column during the adsorption process was calculated according to Equation (3) as follows:

(3) $$q_{e}=\frac{q_{\mathrm{total}}}{m}$$

where *m* (g) is the dry mass of the cryogel. The total quantity of phosphate (*m*_total_; mg) entering the column can be calculated using Equation (4):

(4) $$m_{\mathrm{total}}=\frac{Q\cdot C_{0}\cdot t_{\mathrm{total}}}{1000}$$

The total percentage of adsorption, which is related to column performance, was calculated from the ratio of the total mass of adsorbate (*q*_total_) to the total quantity of phosphate (*m*_total_) [47] using Equation (5):

(5) $$\%\mathrm{Total}\;\mathrm{adsorption}=\left(\frac{q_{\mathrm{total}}}{m_{\mathrm{total}}}\right)\times 100$$

### 2.6. Real Sample Application

A real water sample was collected from the discharge effluent of the municipality treatment plant in Patong, Phuket. Its phosphate concentration was 3.38 mg L^−1^, which exceeded the level that is regarded to cause water eutrophication (>0.02 mg L^−1^) [7]. Thus, the continuous flow adsorption was investigated using the influent flow rate of 10 mL min^−1^ and an adsorbent height of 2 cm.

## 3. Results and Discussion

### 3.1. Characterization of the Adsorptive Material

**Figure 2:**
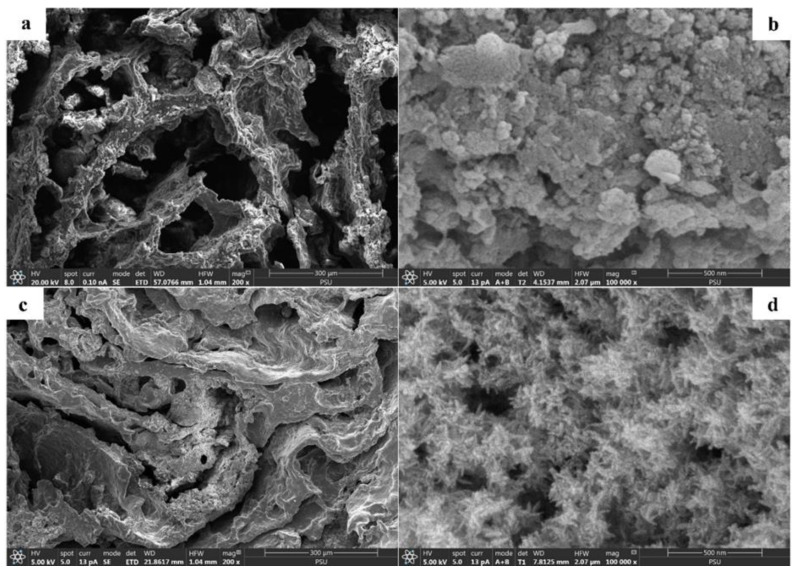
SEM images of Cry–CSH before (**a**,**b**) and (**c**,**d**) after adsorption of phosphate with magnification of 200 (**a**,**c**), and 100,000× (**c**,**d**).

Representative FESEM images of the monolithic Cry–CSH column before and after phosphate adsorption are shown in Figure 2. Both images revealed the presence of macropores in an interconnected polymer network containing crystalline CSH nanoparticles within the walls (Figure 2a,b shows 200 and 100,000×, respectively). Meanwhile, the images of Cry–CSH after phosphate adsorption at the exhaustion time showed particles of calcium phosphate (Figure 2c) comparable to those previously described [10,35]. The acicular structure observed in Figure 2d indicates the presence of hydroxyapatite after phosphate adsorption at the exhaustion time. It has been reported that the spherical particles of amorphous calcium phosphate generated on the CSH surface after phosphate adsorption begin to transform into acicular structures of hydroxyapatite after 8 h and are completely transformed after 24 h [10,35].

**Figure 3:**
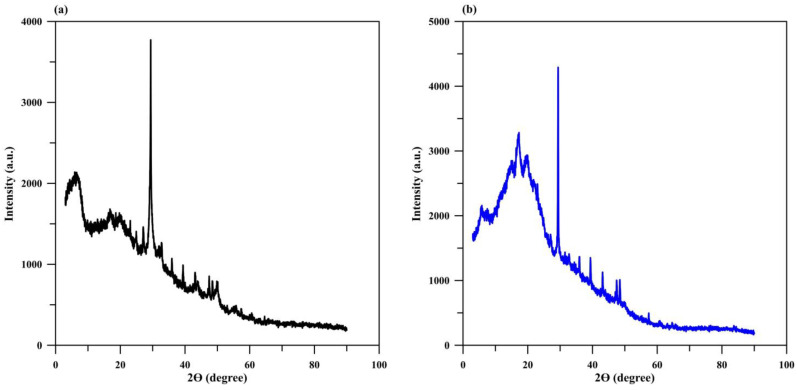
XRD patterns of Cry–CSH (**a**) before and (**b**) after phosphate adsorption.

The XRD patterns of the monolithic Cry–CSH column before (Figure 3a) and after phosphate adsorption (Figure 3b) are quite similar, showing broad humps with a sharp peak at 2θ~13.2° that indicates the amorphous structure of starch cryogel along with some V-type crystalline starch [10]. The characteristic diffraction peaks of CSH were also observed at 2θ~29.1°, which indicates the relatively ordered structure of CSH [10,35,48]. As the acicular structure of hydroxyapatite was observed in the FESEM image after phosphate adsorption at the exhaustion time, the intensity of peak at 2θ~29.1° was expected to decrease or disappear, with a broad peak developing at 2θ~32° due to the transformation of amorphous calcium phosphate to hydroxyapatite [35]. However, the intensity of the peak at 2θ~29.1° increased without any change in the peak at 2θ~32°. This resembled the result that was obtained when the material was analyzed after 2 h of batch adsorption [10]. This phenomenon is the same as that reported in an earlier study stating that no visible product was detected in the XRD pattern, although the efficiency of phosphate recovery by CSH had reached 83.6% and the FTIR spectra confirmed the presence of phosphate on CSH [35]. The transformation of hydroxyapatite may remain incomplete at the exhaustion time of the Cry–CSH column due to the entrapping of CSH nanoparticles within the starch matrix that could interrupt the reaction between phosphate and CSH [10]. This leads to slow completion of the transformation, compared with CSH nanoparticles (24 h) [35], and leads to unchanged XRD patterns.

**Figure 4:**
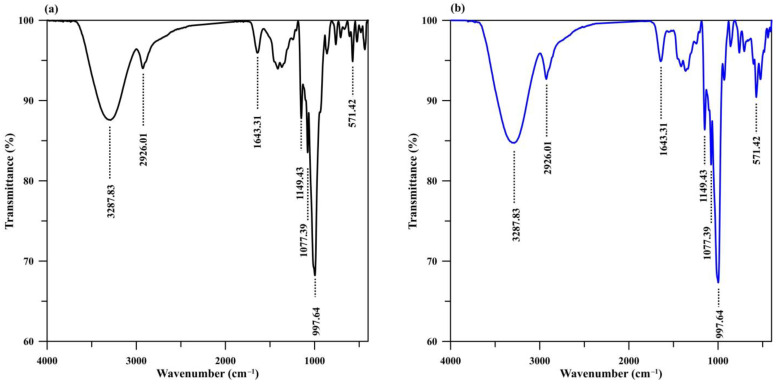
FTIR spectra of Cry–CSH (**a**) before and (**b**) after phosphate adsorption.

The FTIR spectra of the Cry–CSH column before and after phosphate adsorption are displayed in Figure 4. The O–H stretching absorption band of hydroxyl groups in starch and water molecules [10] remains in position at 3288 cm^−1^ both before and after phosphate adsorption. Similarly, the C–H stretching absorption bands in starch molecules remain in position at 2926 cm^−1^ [10]. The absorption band at 1643 cm^−1^ was attributed to H–O–H bending in water molecules in CSH which may overlap with C–O bending in amylopectin in starch [10,49,50]. The remaining intense band at ~998 cm^−1^ before and after phosphate adsorption was attributed to O–Si–O stretching vibrations from CSH molecules [10,34], which overlap with C–O–H bending in amylopectin [49,50,51]. The highly intense band of Cry–CSH after adsorption at ~1077 cm^−1^ can be attributed to the C–O, C–C, and O–H bond stretching bands of starch [49,50,51] that may overlap with the vibration bands of H_2_PO_4_^–^ and HPO_4_^2–^ commonly appearing at 1038 cm^−1^ [33] or 1046 cm^−1^ [35].

### 3.2. Continuous Flow Adsorption Studies

As Cry–CSH is a monolithic composite material that contains a highly interconnected macroporous network with good mechanical strength and very low flow resistance to liquids, it was expected to be applicable to a continuous flow system for the removal of phosphate, which is interesting in terms of automation and large-scale applications. In this study, the parameters that influence the system performance, i.e., initial concentration, flow rate, and column height, were investigated.

#### 3.2.1. Effect of the Influent Flow Rate

**Figure 5:**
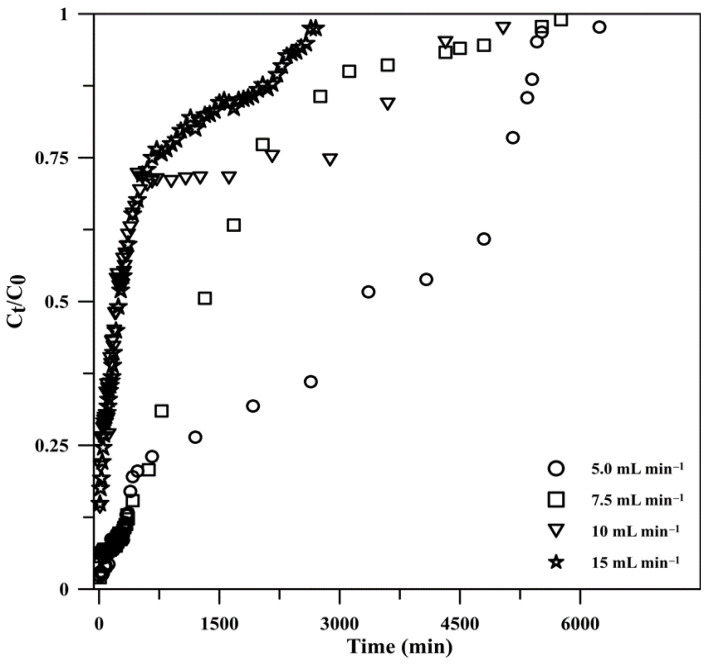
Breakthrough curves for phosphate adsorption onto monolithic Cry–CSH at different flow rates (*C*_t_/*C*_0_ is the ratio of effluent phosphate concentration at time *t* to the influent phosphate concentration).

In a continuous flow system, the influent flow rate is a critical parameter that has a considerable impact on column performance. The flow rate of the influent was investigated from 5.0 to 15 mL min^−1^ for an adsorbent height of 1 cm and a constant phosphate concentration of 2 mg L^−1^ in the influent at room temperature. The experimental breakthrough curves obtained at various influent flow rates showed different breakthrough slopes due to differences in the retention process (Figure 5). The phosphate adsorption was initially extremely rapid probably because of the availability of active sites on the composite and became less effective in the next stage as the sites were gradually occupied. The monolithic composite material could accumulate phosphate even after breakthrough, albeit with lower efficiency. The curves became steeper with increasing the flow rate because the adsorbent was saturated earlier, and the column was exhausted rapidly [52]. As shown in Table 1, the time required to reach the breakthrough and exhaustion states at high feed flows was reduced, as previously described [53]. The mass transfer rate also increased with the flow rate, increasing the amount of phosphate adsorbed on the composite and, thus, leading to faster saturation [54]. The phosphate removal efficiency increased marginally with the flow rate from 5 to 7.5 mL min^−1^ (97.75% to 98.02%) but decreased from 97.75% to 85.15% for a further increase in the flow rate from 10 to 15 mL min^−1^ due to the weakening of the interactions between the adsorbate and adsorbent. Meanwhile, the equilibrium uptake was not influenced by the flow rate (Table 1). The adsorption capacity increased from 15.9 to 29.5 mg g^−1^ upon increasing the phosphate solution flow rate from 5.0 to 7.5 mL min^−1^, followed by decreases to 22.8 and 18.0 mg g^−1^ at 10 and 15 mL min^−1^, respectively. This trend can be explained by the fact that at high flow rates, the amount of phosphate ions in the influent was high, and phosphate was loaded into the column within a short time. As a result of the insufficient contact time and stronger competition among the numerous adsorbate molecules for the limited active sites of the adsorbent, some of the phosphate ions left the column before reaching the adsorption equilibrium [55].

#### 3.2.2. Effect of the Influent Phosphate Concentration

**Figure 6:**
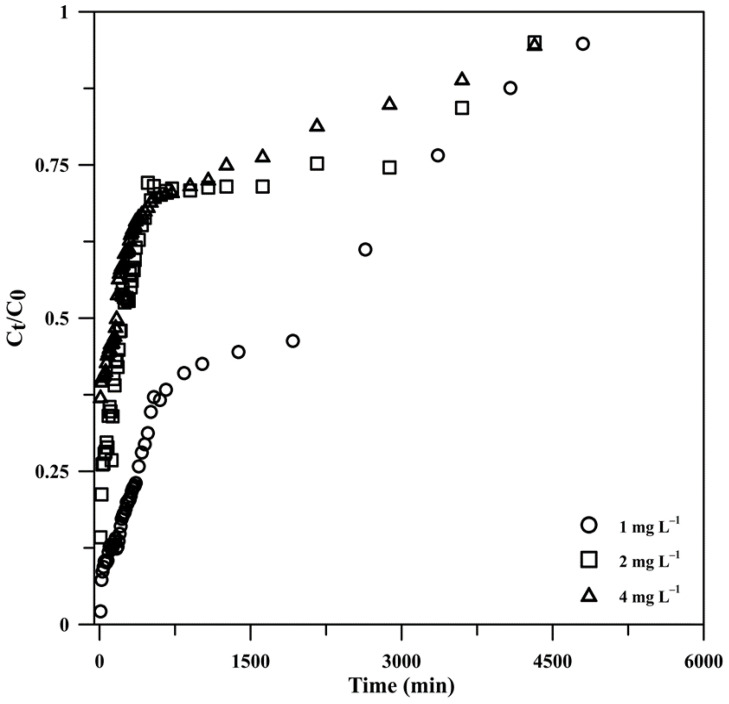
Breakthrough curves for phosphate adsorption onto Cry–CSH at different phosphate concentrations (*C*_t_/*C*_0_ is the ratio of effluent phosphate concentration at time *t* to the influent phosphate concentration).

In continuous flow systems, the initial influent concentration is a limiting factor as a given mass of adsorbent can only absorb a specific quantity of adsorbate. Figure 6 illustrates the breakthrough curves obtained at initial concentrations of phosphate in the feed solution ranging from 1 to 4 mg L^−1^, a constant adsorbent height of 1 cm, a flow rate of 10 mL min^−1^, and room temperature. The breakthrough curve was flatter at lower initial phosphate concentrations, indicating that a relatively large mass transfer zone was formed at the column front where phosphate adsorption occurred. This may contribute to slowing down the saturation of the fixed active sites of the composite, resulting in longer breakthrough times (20 min at 1 mg L^−1^), due to the lower adsorbate dose and the slower transport of phosphate ions stemming from the smaller concentration gradient of phosphate ions on the adsorbent and in the bulk fluid and, thus, from a lower diffusion coefficient [44,54,56]. In contrast, at higher initial concentrations, the adsorption sites were more rapidly covered, leading to a decrease in the breakthrough times (<10 min) and a sharpening of the breakthrough curves, suggesting a relatively smaller mass transfer zone. Therefore, as observed in a previous report [53], the influent volume that a fixed mass of Cry–CSH can purify increases as the initial concentration of phosphate in the influent decreases (48,000 mL at 1 mg L^−1^). The adsorption capacity of phosphate on the column was found to increase from 12.3 to 28.3 mg g^−1^ with increasing the initial phosphate concentration from 1 to 4 mg L^−1^ (Table 2). This may be explained by the high influent phosphate content, which gives the transfer process more motivation to overcome the mass transfer barrier [54,57,58]. However, the adsorption efficiency decreased from 97.85% to 62.74% with increasing the initial concentration because all the phosphate ions in the solution at lower initial concentrations could interact with the fixed binding sites of the composite, which were only marginally saturated, resulting in higher adsorption. In contrast, the increased saturation of the binding sites caused more phosphate ions to stay in the solution at higher initial concentrations, which reduced the effectiveness of the adsorption [54,58].

#### 3.2.3. Effect of the Adsorbent Height

**Figure 7:**
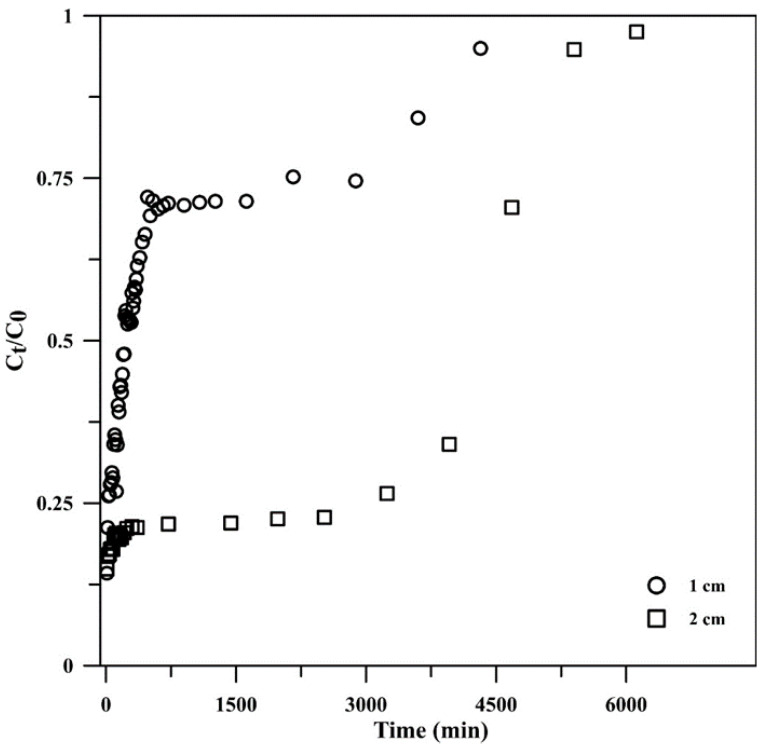
Breakthrough curves for phosphate adsorption onto monolithic Cry–CSH at different adsorbent heights (*C*_t_/*C*_0_ is the ratio of the effluent phosphate concentration at time *t* to the influent phosphate concentration).

The height of the column is correlated with the number of active sites that can accumulate phosphate ions. Thus, it affects the adsorption capacity and efficiency of the column, which are reflected in the steepness of the breakthrough curve. The breakthrough curves were constructed at adsorbent heights of 1 and 2 cm while operating at a constant feed flow rate of 10 mL min^−1^ and an influent phosphate concentration of 2 mg L^−1^ (Figure 7). The results showed that exhaustion times increased with adsorbent height. Thus, the contact times between the adsorbate and the adsorbent became longer, resulting in a decline in the breakthrough slope [55]. The increase in the column height increases the mass transfer zone in the column [59,60], which moves from the entrance toward the exit. For this reason, if the flow rate and influent concentration remain the same, an increase in the adsorbent height will cause the mass transfer zone to travel further before reaching the exit, leading to a flatter breakthrough curve and longer breakthrough and exhaustion periods [5]. The total phosphate uptake increased from 74.12 to 104.03 mg with increasing the column height from 1 to 2 cm (Table 3) owing to an increase in the composite’s surface area, which improved the availability of phosphate-binding sites during the adsorption process [54]. The phosphate adsorption efficiency remained virtually unaltered (85.79% and 85.15%) upon increasing the column height, whereas the treated volume increased from 43,200 to 63,200 mL.

### 3.3. Adsorption Dynamics

In this work, the Thomas, Adams–Bohart, and Yoon–Nelson models were used to study the behavior of the phosphate uptake onto the monolithic Cry–CSH column.

#### 3.3.1. Thomas Model

One of the most general and widely used methods in column performance theory is the Thomas model [61], which is proposed assuming a Langmuir adsorption isotherm and that the rate driving force obeys second-order reversible reaction kinetics [62]. The model is based on the hypothesis that the adsorption is limited by the mass transfer at the interface instead of by chemical interactions between molecules [5,63], and the corresponding linear equation is expressed as [46]:

(6) $$\ln\left(\frac{C_{0}}{C_{t}}-1\right)=\frac{k_{\mathrm{TH}}q_{e}m}{Q}{-k}_{\mathrm{TH}}C_{0}t$$

where *C*_0_ and *C*_t_ represent the influent and effluent phosphate concentrations, respectively, *k*_TH_ (mL min^−1^ mg^−1^) is Thomas rate constant, *t* (min) is the total flow time, *q*_e_ (mg g^−1^) is the predicted adsorption capacity, *m* (g) is the mass of the adsorbent within the column, and *Q* (mL min^−1^) is the flow rate.

By plotting $\ln\left(\frac{C_{0}}{C_{t}}-1\right)$ versus *t*, the values of *k*_TH_ and *q*_e_ were determined from the slope and the intercept of the linear plot, respectively, and the results are summarized in Table 4. The *k*_TH_ value increased from 0.50 to 0.90 mL min^−1^ mg^−1^ with increasing the flow rate from 5 to 15 mL min^−1^, whereas *q*_e_ showed the reverse trend. This is due to an insufficient residence time for phosphate diffusion into the cryogel pores, which resulted in phosphate leaving the column before the equilibrium was reached [54]. The *k*_TH_ value decreased upon increasing the initial concentration similarly to when the height of the cryogel increased. These results were in good agreement with previous reports [47,54]. The increased *k*_TH_ with increasing the flow rate and decreased *k*_TH_ with increasing the inlet concentration and adsorbent height suggested that external mass transfer dominated the overall system dynamics [54]. These trends indicate that the overall system kinetics were dominated by external mass transfer [54].

#### 3.3.2. Adams–Bohart Model

The Adams–Bohart model [64] is appropriate for explaining the initial part of the adsorption breakthrough curve, where *C*_t_/*C*_0_ is lower than 0.5. The linear equation of this model is expressed as:

(7) $$\ln\left(\frac{C_{t}}{C_{0}}\right){=k}_{\mathrm{AB}}C_{0}{t-k}_{\mathrm{AB}}C_{s}\left(\frac{z}{U_{0}}\right)$$

where *k*_AB_ (L mg^−1^ min^−1^) is the Adams–Bohart rate constant or mass transfer coefficient, *C*_s_ (mg L^−1^) is the saturation concentration, *z* (cm) is the bed height, and *U*_0_ (cm^−1^ min) is the superficial velocity calculated by dividing *Q* (mL^−1^ min) by the cross-sectional area of the column (A; cm^2^) [65,66,67].

The *k*_AB_ and *C*_s_ parameters were determined by plotting $\ln\left(\frac{C_{t}}{C_{0}}\right)$ against *t* for *C*_t_/*C*_0_ < 0.5, finding that *k*_AB_ increased with increasing the influent flow rate but decreased when the initial phosphate concentration and adsorbent height increased (Table 4). This revealed that external mass transfer dominated the overall system dynamics during the initial stage of phosphate adsorption on the cryogel column [43,58]. Meanwhile, the *C*_s_ value declined as the flow rate increased but enhanced with the initial concentration of phosphate and adsorbent height. The correlation coefficients (*R^2^*) were higher for the Adams–Bohart model than for the Thomas model; however, the validity of the former is limited to the range of conditions used.

#### 3.3.3. Yoon–Nelson Model

The Yoon–Nelson model [68] is a theoretical model utilized for a single-component system and does not focus on the adsorbate characteristics, type of adsorbent, or any physical features of the adsorption bed. This model assumes that the decreasing adsorption rate is proportional to both the adsorbate adsorption and the breakthrough on the adsorbent as expressed by the linearized equation:

(8) $$\ln\left(\frac{C_{t}}{C_{0}-C_{t}}\right){=k}_{\mathrm{YN}}t-\tau k_{\mathrm{YN}}$$

where *k*_YN_ (min^−1^) is the Yoon–Nelson rate constant and *τ* (min) is the time required for 50% adsorption breakthrough. The values of *k*_YN_ and *τ* were determined by plotting $\ln\left(\frac{C_{t}}{C_{0}-C_{t}}\right)$ versus time (t). The *k*_YN_ value increased with increasing the influent flow rate and the initial phosphate concentration but decreased with the adsorbent height. The 50% breakthrough times significantly decreased when the influent flow rate and the initial phosphate concentration increased because the column saturated faster [69].

Considering the *R^2^* values of the three theoretical models summarized in Table 4, the Thomas and Yoon–Nelson models provided similar results, whereas the experimental data fitted better to the Adams–Bohart model at the initial stage of the adsorption process.

### 3.4. Application of the Continuous Flow System for Real Sample Treatment

**Figure 8:**
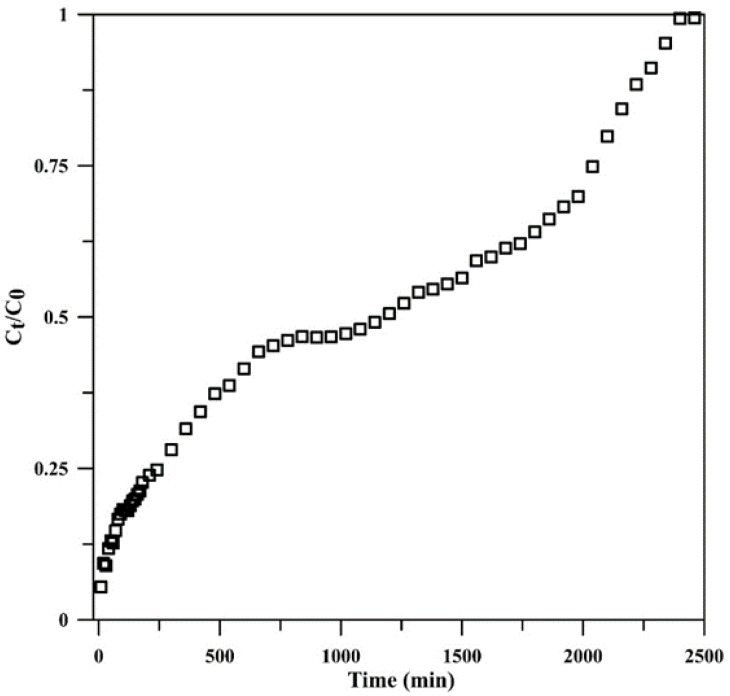
Breakthrough curve for phosphate adsorption by monolithic Cry–CSH using a real sample.

The developed continuous flow system using a cryogel monolithic column was applied for phosphate removal from a real sample with a phosphate concentration of 3.38 mg L^−1^, achieving a phosphate removal of 94.61%. The breakthrough curve for wastewater is shown in Figure 8.

These results demonstrate that a monolithic Cry–CSH column can be used for the continuous flow adsorption of phosphate without any loss of active material, although its removal capacity may be lesser than other composites (Table 5).

## 4. Conclusions

The adsorption of phosphate on a monolithic Cry–CSH adsorbent was evaluated for the first time using a continuous flow system. The flow rate of phosphate in the influent, the initial phosphate concentration, and the height of the adsorbent affected the adsorption performance and the characteristics of the breakthrough curve. Because the adsorbent’s surface is rapidly saturated with phosphate ions at a higher flow rate and initial phosphate concentration, the breakthrough and exhaust times are decreased. On the contrary, the increase in adsorbent height significantly improved the performance of the column by increasing the breakthrough and exhaustion times of the adsorption process.

The experimental data were fit to the Thomas, Adams–Bohart, and Yoon–Nelson models, allowing for an assessment of the breakthrough curves and the determination of the column’s characteristic properties for use in process design. Both the Thomas and Yoon–Nelson models provided similar results, whereas the experimental data fitted better to the Adams–Bohart model at the initial stage of the adsorption. Overall, the results suggested that moderate flow rates, low initial phosphate concentration, and high adsorbent height are beneficial to the column efficiency in phosphate removal for extended operation times. Moreover, the continuous flow system was applied to remove phosphate from the discharge effluent of the municipality treatment plant in Patong, Phuket (3.38 mg L^−1^), achieving removal of 94.61%. Therefore, this study demonstrates that the Cry–CSH monolith is a promising adsorbent for application in sustainable phosphate removal from water.

## Figures and Tables

**Table 1:** Influence of the solution flow rate on experimental breakthrough parameters for phosphate adsorption by monolithic Cry–CSH.

*Q* (mL min^−1^)	*C*_0_ (mg L^−1^)	*z* (cm)	*t*_b_ (min)	*t*_total_ (min)	*V*_eff_ (mL)	*m*_total_ (mg)	*q*_total_ (mg)	*q*_e_ (mg g^−1^)	%Total Adsorption
5.0	2	1	60	6240	31,200	62.40	61.00	15.93	97.75
7.5	2	1	20	5760	57,600	115.20	112.92	29.48	98.02
10	2	1	<10	5040	50,400	100.80	86.48	22.58	85.79
15	2	1	<10	2700	40,500	81.00	68.97	18.01	85.15

**Table 2:** Influence of the phosphate initial concentration on experimental breakthrough parameters for phosphate adsorption by monolithic Cry–CSH.

*Q* (mL min^−1^)	*C*_0_ (mg L^−1^)	*z* (cm)	*t*_b_ (min)	*t*_total_ (min)	*V*_eff_ (mL)	*m*_total_ (mg)	*q*_total_ (mg)	*q*_e_ (mg g^−1^)	%Total Adsorption
10	1	1	20	4800	48,000	48.00	46.97	12.26	97.85
10	2	1	<10	4320	43,200	86.40	74.12	19.35	85.79
10	4	1	<10	4320	43,200	172.80	108.42	28.31	62.74

**Table 3:** Influence of column height on experimental breakthrough parameters for phosphate adsorption by monolithic Cry–CSH.

*Q* (mL min^−1^)	*C*_0_ (mg L^−1^)	*z* (cm)	*t*_b_ (min)	*t*_total_ (min)	*V*_eff_ (mL)	*m*_total_ (mg)	*q*_total_ (mg)	*q*_e_ (mg g^−1^)	%Total Adsorption
10	2	1	<10	4320	43,200	86.40	74.12	19.35	85.79
10	2	2	<10	6120	61,200	122.40	104.23	13.61	85.15
10	RW * (3.38)	2	10	2460	24,600	83.15	78.67	10.27	94.61

* RW: A real water sample collected from effluent discharged from the Patong Municipality Treatment Plant, Phuket, Thailand.

**Table 4:** Estimation of dynamic model parameters for phosphate adsorption onto monolithic Cry–CSH.

	Thomas			Adams–Bohart			Yoon–Nelson		
	*k*_TH_ (mL mg^−1^ min^−1^)	*q*_e_ (mg g^−1^)	*R^2^*	*k*_AB_ (L^−1^ mg min)	*C*_s_(mg L^−1^)	*R^2^*	*k*_YN_ (min^−1^)	*τ* (min)	*R^2^*
***Q* (mL min^−1^)**									
5.0	0.50	7.60	0.9026	0.0005	1428	0.8634	0.0009	2990	0.9043
7.5	0.65	7.82	0.9550	0.0011	1018	0.9566	0.0013	1998	0.9550
10	0.80	1.89	0.6227	0.0018	425	0.9551	0.0013	379	0.6227
15	0.90	3.96	0.8701	0.0020	625	0.9411	0.0014	453	0.8849
***C*_0_ (mg L^−1^)**									
1	1.50	3.39	0.8267	0.0030	421	0.9562	0.001	1847	0.8267
2	0.80	1.89	0.6227	0.0018	425	0.9551	0.0013	379	0.5311
4	0.25	0.11	0.6593	0.0004	1225	0.9591	0.0015	92	0.7221
***z* (cm)**									
1	0.80	1.89	0.6227	0.0018	425	0.9551	0.0013	379	0.5311
2	0.30	7.19	0.7668	0.0007	707	0.7172	0.0006	2754	0.7668
RW ^1^	0.53	4.53	0.8259	0.0006	854	0.8701	0.018	103	0.8259

^1^ RW: A real water sample collected from effluent discharged from the Patong Municipality Treatment Plant, Phuket, Thailand.

**Table 5:** Comparison of Cry–CSH column with various adsorbents for phosphate removal.

Adsorbent	Adsorption System	Removal Capacity (mg PO_4_^3–^/g)	Real Sample Application	Removal Efficiency in Real Sample	Problem	Reference
MCM-41	Batch	21.01	No	-	Loss of material	[7]
C–S–H	Batch	109.4	No	-	Loss of material	[35]
Cry-CSH	Batch	64.52	Yes	98.6% to 99.8%	No loss	[10]
PVA-CSH	Batch/fixed bed column	28.15	Yes	Not reported	Loss of material	[34]
Am-Zr/MgFe *	Fixed bed column	25.15	Yes	Not reported	Loss and clog	[53]
HFeO **	Fixed bed column	53.57	No	-	Loss and clog	[55]
AdBg ***	Fixed bed column	4.18	Yes	6.69 mg/g	Loss and clog	[65]
Cry-CSH	Monolithic column	13.61	Yes	94.61%	No loss/no clog	This work

* Am-Zr/MgFe: Zirconium (hydr)oxide/MgFe layered double hydroxides composite; ** HFeO: an anion exchange resin impregnated with iron hydroxide oxides; *** AdBg: 50% Andosol-bagasse mixture.

## Data Availability

Not applicable.
